# Managing alcohol-related attendances in emergency care: can diversion to bespoke services lessen the burden?

**DOI:** 10.1136/emermed-2016-206451

**Published:** 2017-11-28

**Authors:** Andy Irving, Steve Goodacre, Joanne Blake, Davina Allen, Simon C Moore

**Affiliations:** 1 Centre for Urgent and Emergency Care Research (CURE), School of Health and Related Research (ScHARR), University of Sheffield, Sheffield, UK; 2 School of Healthcare Sciences, Cardiff University, Cardiff, UK; 3 School of Dentistry, Cardiff University, Cardiff, UK

**Keywords:** alcohol abuse, emergency care systems, emergency departments, emergency department utilisation, prehospital care, clinical management

## Abstract

Acute alcohol intoxication (AAI) has a long history of burdening emergency care services. Healthcare systems around the world have explored a variety of different services that divert AAI away from EDs to better manage their condition. Little formal evaluation has been undertaken, particularly in the UK where alcohol misuse is one of the highest in the world. In this article, we outline a brief history of diversionary services, introduce the concept of Alcohol Intoxication Management Services (AIMS) and describe examples of AIMS in the UK. We then describe Evaluating the Diversion of Alcohol-Related Attendances, a natural experiment including six cities with AIMS compared with six cities without, that involves an ethnographic study, records patient experiences in both AIMS and EDs, assesses impact on key performance indicators in healthcare and evaluates the cost-effectiveness of AIMS.

## Introduction

EDs across the world are familiar with alcohol-related attendances, especially acute alcohol intoxication (AAI)^1^ and related injury.^2^ Estimates on the number of intoxicated patients attending ED vary from 4.5% of all attending patients in Australia to 7.9% in North America, and 12**–**15% in the UK.^3–7^ The effects of AAI are not limited to the patient. They are a burden to and disruptive influence on the emergency healthcare system and its patients generally. In the UK, alcohol-related ED attendances peak on Friday and Saturday evenings when up to 70% of all attendances can be alcohol related.^4 6 8^ Many AAIs arrive by ambulance (40% in the UK^6^) and on arrival they are typically triaged into the more resource-intensive ‘majors’ route, leaving less urgent patients waiting longer to be seen.^9–13^ While intoxication is far from being a new phenomenon internationally or in the UK**—**one of the heaviest drinking regions in the world^14^ **—**UK EDs are increasingly examining ways to better manage AAI while waiting for the prevention agenda to gain traction. One notable development in the UK has been to implement bespoke services that are able to divert and safely manage AAI away from ED: Alcohol Intoxication Management Services (AIMS), but also variously named Drunk Tanks, Safe Havens and Alcohol Treatment Centres (ATCs). There is increasing interest in the use of AIMS to divert AAI away from the ED but no evidence until now that UK AIMS are effective.

While alcohol has been used since at least 7000 BC, the earliest records for services similar to AIMS are found in Russia in the 18th century. Rooms were allocated for sobering-up in the quarters of at least one of Russia’s cavalry regiments.^15 16^ However, it was economic concerns over the number of workers who became intoxicated, fell over and died of hypothermia in the Moscow winter that motivated the implementation of vytrezvitels (sobering-up stations) in 1902.^15^ By 1959, the number of sobering-up stations, distinct from specialised psychiatric facilities for alcoholics, in Russia was increasing but attracted criticism for not being sufficiently integrated with social facilities and for low standards in the care offered.^16^ In Europe, at least four countries appear to offer services that divert uncomplicated intoxications away from ED into specialised ‘sobering-up stations’, including the Czech Republic, Poland, Austria and Finland.^17 18^ In North America. police run ‘drunk tanks’^19 20^ were gradually replaced with specialised services in response to the 1971 Uniform Alcoholism and Intoxication Treatment Act, which shifted the definition of simple intoxication away from criminal justice to public health.^21^ A variety of diversionary services for AAI emerged in Washington DC,^19 21^ Massachusetts, San Diego^22^ and San Francisco.^23^ In total, across the USA, there are at least 27 short-term (≤2 nights) sobering-up centres providing an alternative to custody or ED in operation.^24^ Similar services are apparent in Canada (Toronto, Ottawa and Calgary^25^). In Australia, ‘sobering-up centres’ were established during the 1980s as an alternative to the custodial response to public intoxication.^26^ New Zealand also offers similar services, but only for specific events such as festivals.^17 27^

While service provision varies considerably around the world, there is a scarcity of formal evaluation. The limited evaluations undertaken suggest that diversionary services can reduce hospital attendance due to AAI by up to 50% in the example of San Diego,^25^ while in San Francisco, there is evidence that the service successfully diverts patients away from ED and provides social assistance and continuity of care to the most vulnerable.^23^ There have been no evaluations in the UK and this is in spite of AIMS becoming increasingly common in locations characterised by a high density of bars and nightclubs.^28^ The Evaluating the Diversion of Alcohol-Related Attendances (EDARA) project aims to address this knowledge gap.

## Diversion services in the UK: AIMS

AIMS are designed to deal with the problem of AAI by offering an alternative care pathway within the urgent and emergency care system. They therefore need to be open at predictable times in the week and in locations associated with a high incidence of AAI. Our definition of AIMS is a sustained and regular service open to the general public and not sporadic services or ‘pop up’ single event facilities only for event attendees. This definition clearly delineates AIMS from other alcohol/substance misuse community or hospital-based interventions for people with alcohol use disorders and other complex long-term problems with substances.

There is evidence that AIMS have been in existence in the UK since at least 2000. Services are diverse, ranging from the Cardiff ATC (box 1) to less clinical services such as the Manchester Safe Haven (box 2). Nevertheless, there are common features:

- Initiated by local agencies that identify the problems and impacts AAI is having on services and on the wider community.
- Set up and run by multiple agencies, for example, ambulance services, local hospital trusts, police, local councils and voluntary sector organisations, so staffing can be a mix of nurse, paramedic, police and volunteers.
- Located in buildings or mobile units stationed in or near to areas with a high density of licensed premises.
- Open regularly and at times when AAI is expected to peak (eg, Friday and Saturday evenings) but may offer additional cover on other evenings such as student nights, sporting events and national holidays.
- Users are typically referred in by local ambulance, police or third sector volunteers (eg, Street Pastors).
- Users would typically be triaged by a suitably qualified person, given basic first aid, water and monitored while they sober up.
- Users may be screened for alcohol use problems and offered some form of advice, brief intervention or referral for specialist alcohol support.
- Users are discharged at the point at which they are deemed safe, usually to the care of friends or family, with plans for safe travel home discussed and arranged.

## Can patients with AAI be safely diverted away from the ED?

Evidence suggests that EDs may see more AAIs than are warranted, possibly due to risk aversion in referring agencies or just because there is nowhere else for the very intoxicated to go at night.^29^ Evidence from several studies suggest that a proportion of patients attending ED due to AAI may be over-medicalised and could receive care in facilities other than ED. Emergency medical technicians in Rhode Island were able to accurately discriminate between patients who required emergency attention and intoxicated patients who would be suitable for diversion based on a comparison of their assessment with patient outcomes in ED.^30^ A similar study in San Francisco and with paramedics found that potentially 28% of intoxicated patients who are not expected to require emergency attention could be diverted away from the ED.^31^ Similarly, in Colorado, an evaluation of a nurse-led facility found 0.6% who were diverted away from ED subsequently required referral to ED and 58% referred to ED from the nurse-led facility required emergency attention.^32^

## Evaluating the diversion of alcohol-related attendances

EDARA builds on pilot work in Cardiff. Cardiff is a medium-sized city in the UK, it has a resident population of approximately 360 000, but it is also a destination city for drinkers. The bars, clubs and other licensed venues in Central Cardiff have a total capacity of more than 100 000. Accordingly, the local ED had become besieged with AAI and in response the Cardiff ATC (see box 1) was developed in 2012. The pilot evaluation suggested that it had successfully diverted patients away from the ED, but also suggested that the additional capacity provided by the AIMS was associated with an increase in demand for health services, possibly due to pre-existing unmet need in the night time economy.^29^ A need for a controlled evaluation was identified.

EDARA is a large mixed-method multicentre study funded by the UK National Institute for Health Research to evaluate the impact of UK AIMS. The project evaluates the acceptability, effectiveness and cost-effectiveness of AIMS. It compares UK cities in which AIMS have been implemented with control cities without AIMS. Lack of experimental control over the implementation of the intervention presents a common challenge in health services research and as such EDARA is in the form of a natural experiment.

An ethnographic component uses a mixture of observational work and conversations with staff to explore how intoxicated patients impact the working lives (eg, job role, morale, stress, occupational identity and job satisfaction) of frontline staff, including police, ambulance, healthcare professionals and affiliated volunteers. This enables EDARA to gain a good understanding of how different agents work together to manage and care for intoxicated individuals. By focusing on areas with (two cities) and without (one city) AIMS, the ethnographic component will elucidate the impact of AIMS and explore the barriers and facilitators to AIMS implementation. It will explore the views of intoxicated individuals using AIMS, the views of people using EDs (as they may benefit through reducing the number of intoxicated people in shared waiting areas) in six cities with and six cities without AIMS, analysis of AIMS activity and outcomes, comparison of ambulance service and hospital key performance indicators and simulation modelling of cost-effectiveness. The choice to undertake the ethnographic study with a subset of cities involved in the effectiveness study is pragmatic. To include all six cities would have notably increased either the resources or timeline, neither was feasible.

The primary outcome for the effectiveness analysis is the rate of ED attendance during times of AIMS activity. Other outcomes include hospital admissions and ambulance response times during times of AIMS activity. Cost-effectiveness will estimate the costs of setting up and running each AIMS, and the cost per ED attendance avoided through AIMS availability. The study commenced on 1 January 2016 and is a collaboration between researchers in health services innovation, public health and alcohol-related harm in Cardiff University and researchers from the Centre for Urgent and Emergency Care Research (CURE) at the University of Sheffield.

Given the complexity of the area to be examined, members of three patient and public involvement (PPI) groups are invited to provide advice: (1) The Involving People Network, Wales, (2) The Sheffield Emergency Care Forum and (3) The Sheffield Addiction Recovery Research Panel. The knowledge and experience of the latter two PPI groups were also used to inform the development of surveys for AIMS and ED patients, ensuring they were suitable for the target groups.

In accordance with planned study timeframes, initial ethics approval was granted on 15 April 2016 and the main data collection phase will continue through 2017. The findings are due in 2018 and are intended to provide evidence on whether AIMS should be rolled out as a standard means of managing patients presenting with AAI to the emergency services at times of peak incidence.

## Conclusion

Alcohol intoxication is common around the world, as is the burden they place on emergency care. Diverting these patients away from EDs and into specialised services can alleviate pressures in ED but there is limited evidence of effectiveness, particular so in the UK where alcohol misuse is prominent. EDARA is a large mixed-methods natural experiment that will provide evidence of effectiveness, cost-effectiveness, determine whether patients regard services as acceptable and assess impact on frontline staff involved with managing intoxication.

**Figure 1:**
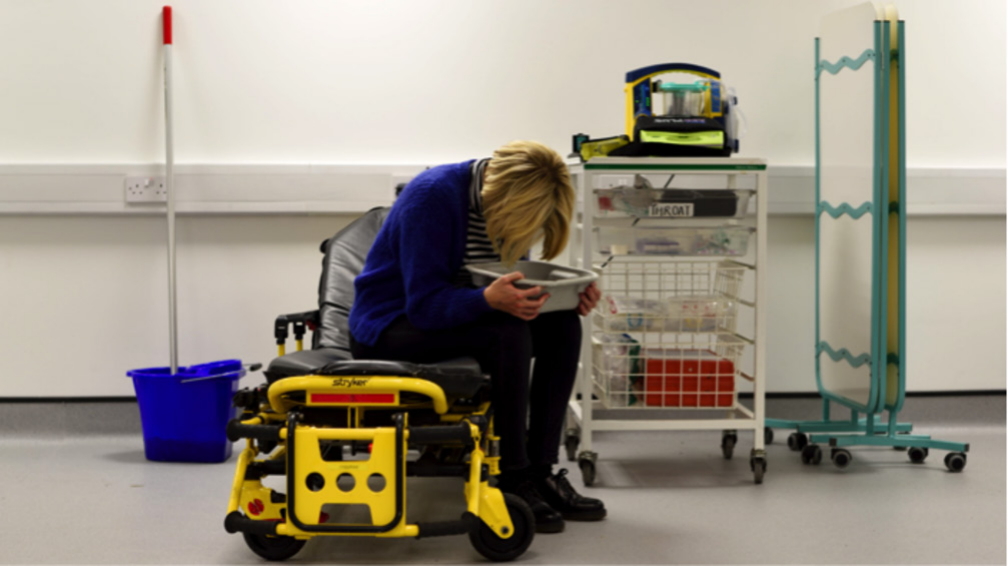
Treatment area of the Cardiff Alcohol Treatment Centre. Cardiff University, reprinted with permission.

**Figure 2:**
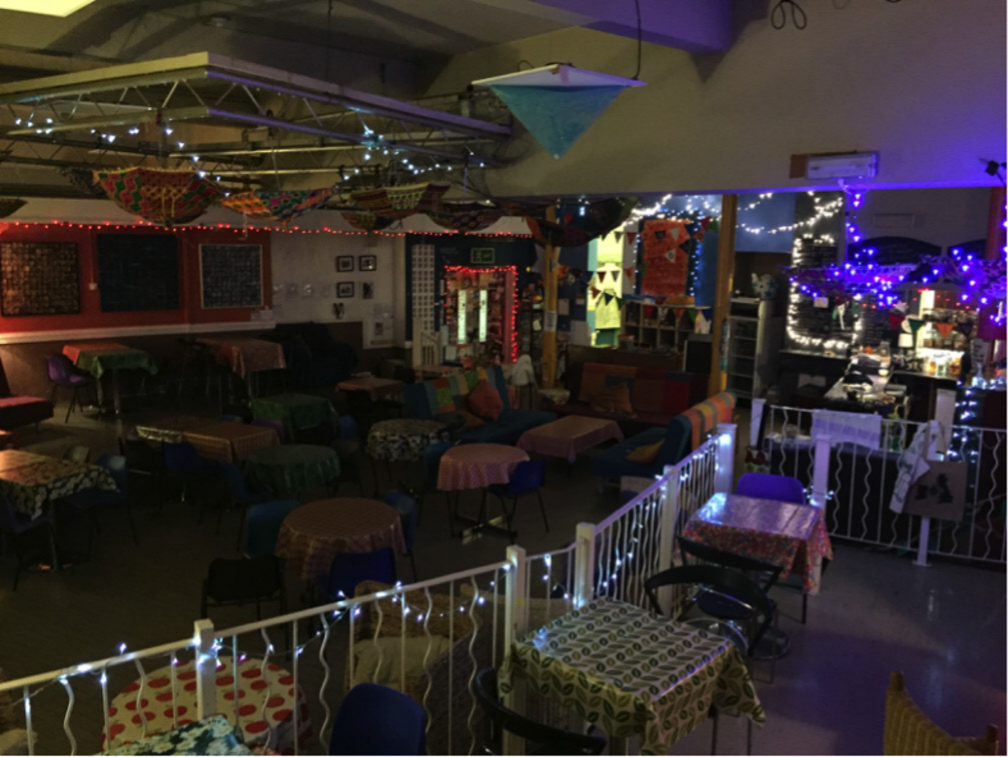
Nexus Art Cafe: Safe Haven.

AIMS can take a number of different forms and vary in structure, staffing, organisation and ability to deliver clinical care. (boxes 1 and 2; figures 1 and 2)

Box 1 **Cardiff Alcohol Treatment Centre (ATC)**

Following a successful 1-year pilot in 2012, funded by the Welsh Government, the Alcohol Treatment Centre (ATC) received further support from the Welsh Government’s Regional Collaboration Fund from 2013 to 2015. The current financial agreement is a joint arrangement between the Cardiff and Vale University Health Board, South Wales Police, the Welsh Ambulance Service and the Cardiff and Vale Area Planning Board. The service is run from a building in Cardiff city centre and is led by a senior nurse practitioner from the University Hospital of Wales emergency department, supported by other nurse practitioners, an urgent care service assistant, a paramedic and police officer. The space includes a large clinical treatment room (partial partitions if needed, figure 1), treatment room with additional diagnostic and treatment resources if required, separate triage assessment and friend/relative waiting area. The service sees on average 10 patients per session with the bulk of referrals brought in by ambulance, followed by those escorted to the ATC by police. AAI is responsible for most attendances but minor injuries due to falls or assaults are in evidence. ATC users are followed up by a phone call from a local substance use support service that offers advice and support around alcohol use and can provide one-to-one counselling, brief interventions and semistructured group work.

Box 2 **Manchester Safe Haven**

Alcohol is a substantial contributor to criminal activity with estimates that 50% of police workload is alcohol related.^33^ A recent UK survey of 4022 police officers found 92% believed alcohol had a large or very large impact on workload policing the night time economy at weekends. Greater Manchester Police identified several issues relating to dealing with perpetrators and victims of crime who are made vulnerable through alcohol use, including a number of incidents in the city centre resulting in fatalities. The Manchester Safe Haven was a police-led initiative funded by the Manchester City Council Community Safety Partnership Team. The service ran from May 2015 to March 2017 and was based in the city centre Nexus Art Café. Café staff and Greater Manchester Police volunteers ran the service on Saturdays from 23:00 to 6:00. The space is a large open plan café with a coffee bar, seating and a sofa ‘relaxation area’ (figure 2). Most referrals were acutely intoxicated individuals found by police in the local area. The Safe Haven provided a space where lone or vulnerable persons were brought in for practical assistance (eg, to charge their mobile phone and to make arrangements for their safe travel home).

Nexus Art Café: Safe Haven
